# QSPR analysis of anticancer drugs using the Euler–Sombor index and theoretical insights on its minimum value for unicyclic graphs

**DOI:** 10.1038/s41598-026-36855-x

**Published:** 2026-02-02

**Authors:** Swathi Shetty, B. R. Rakshith, N. V. Sayinath Udupa

**Affiliations:** Manipal Institute of Technology, Manipal Academy of Higher Education, Manipal, India

**Keywords:** Sombor index, Euler–Sombor index, Anticancer drugs, QSPR analysis, Unicyclic graphs, Chemistry, Mathematics and computing

## Abstract

Topological indices (TIs) are powerful tools for exploring the relationship between molecular structure and drug activity, offering a cost-effective alternative to experimental screening. The Euler–Sombor index, a recently introduced degree-based TI, captures key structural features of molecules that influence their physico-chemical behaviour. This study investigates the use of the Euler–Sombor index in quantitative structure–property relationship (QSPR) modeling of anticancer drugs. By applying linear, quadratic, and logarithmic regression models, we predict important properties such as boiling point (BP), melting point (MP), enthalpy (E), and molar refraction (MR). The results show a significant correlation between the Euler–Sombor index and these molecular properties. In addition, we extend the result of Su and Tang by characterizing unicyclic graphs that have the third minimum Euler–Sombor index.

## Introduction

Quantitative structure–property relationship (QSPR) analysis has emerged as a powerful approach in modern drug discovery, particularly in the study of anticancer agents. The development of new drugs is often expensive, time-intensive, and requires extensive experimental validation, making computational models highly valuable for predicting molecular behaviour. By establishing correlations between molecular structure and physico-chemical properties, QSPR provides a reliable framework to estimate characteristics such as boiling point, melting point, enthalpy, and molar refraction before experimental synthesis^1^. This not only accelerates the identification of drug but also reduces the risk of toxicity and inefficacy in later stages of testing. In the context of cancer treatment, where rapid and effective drug development is crucial, QSPR Modeling based on topological indices offers an efficient tool to understand structure–activity relationships. Structure–property modeling and isomer discrimination through QSPR analysis using topological indices and have been investigated in^2,3^. Recently, Sombor-type topological indices have been extensively applied in QSPR and QSAR studies. In particular, their effectiveness has been demonstrated in modeling physicochemical and biological properties of anticancer drugs, amylose, and polycyclic aromatic hydrocarbons using regression and data-driven approaches^4–6^. For some recent works on topological indices and QSPR Modeling, one can refer^7–11^.

In this article, we assume that *G* is a graph consisting of *n* vertices and *m* edges. We denote its vertex set and edge set as *V*(*G*) and *E*(*G*), respectively. If two vertices *u* and *v* are adjacent, we denote this relationship as $u \sim v$. A pendant vertex is a vertex of degree one. A vertex that is adjacent to a pendant vertex is a quasi-pendant vertex. The class of unicyclic graphs of order *n* and girth *k*, $3\le k\le n$ is denoted as $\mathcal {U}_{n,k}$, and the class of graphs formed by attaching a pendant vertex of a path to a vertex on the cycle $C_{k}$ is denoted as $U_{n,k}$, see Fig. 19. Topological indices are essential tools in chemical graph theory that connect the structure of molecular graphs to various physico-chemical properties of compounds. Numerous vertex-degree-based indices (VBD indices) have been developed in the literature to capture the structural features of molecular graphs. These indices are extensively used in predicting molecular properties and aiding in QSAR/QSPR studies. In recent years, some new VDB indices with geometric interpretations have been discovered. Gutman^12^ proposed a novel method for designing such indices in 2021 and introduced the Sombor index *SO*(*G*). It is given by

$$SO(G) = \sum _{uv \in E(G)} \sqrt{d(u)^2 + d(v)^2},$$

See^13–16^ for details. Gutman et al.^17^ later introduced the Elliptic-Sombor (ESO) index as

$$ESO(G) = \sum _{uv \in E(G)} (d(u) + d(v)) \sqrt{d(u)^2 + d(v)^2}.$$

This index relates to the perimeter of an ellipse and has meaningful chemical interpretations. See^18^ for details.

Recently, Barman and Das^19^, introduced hyperbolic Sombor index *HSO*(*G*). It is defined as

$$HSO(G)=\sum \limits _{uv\in E(G)}\dfrac{\sqrt{d(u)^2 + d(v)^2}}{min\left\{ d(u),d(v)\right\} }.$$

In this context, Gutman^20^ and Tang et al.^21^ independently introduced the Euler–Sombor index *EuSO*(*G*). It is defined as

$$EuSO(G) = \sum \limits _{uv \in E(G)} \sqrt{d(u)^2 + d(v)^2 + d(u)d(v)}.$$

This newly introduced index has attracted attention for its mathematical depth and chemical relevance. Inequalities connecting the Sombor index and Euler–Sombor index were established in^20^, while extremal values for trees, unicyclic, and bicyclic graphs were determined in^21,22^. Further investigations have lead to extremal graphs for unicyclic graphs with fixed girth and diameter^22,23^, as well as minimal and maximal graphs among tricyclic graphs^24,25^. See^26,27^, for additional studies on Euler–Sombor index.

Motivated by these developments, we investigate the use of the Euler–Sombor index in quantitative structure–property relationship (QSPR) modeling of anticancer drugs. By applying linear, quadratic and logarithmic regression models, we predict important properties such as boiling point (BP), melting point (MP), enthalpy (E), and molar refraction (MR). In addition, we characterize unicyclic graphs that have the third minimum Euler–Sombor index.

Extremal topological indices play an important role in QSPR analysis by describing the range within which a physicochemical property can vary for a given class of molecules, such as different isomers or specific graph families. The study of extremal molecular structures helps clarify which structural characteristics have the greatest impact on the property values. In this context, the QSPR analysis presented in the section “QSPR analysis of anticancer drugs” and the theoretical investigation in the section “Unicyclic graph with third minimum Euler–Somborindex” focus on complementary aspects of the Euler–Sombor index, together providing a coherent understanding of both its practical predictive ability and its theoretical bounds.

## QSPR analysis of anticancer drugs

**Fig. 1:**
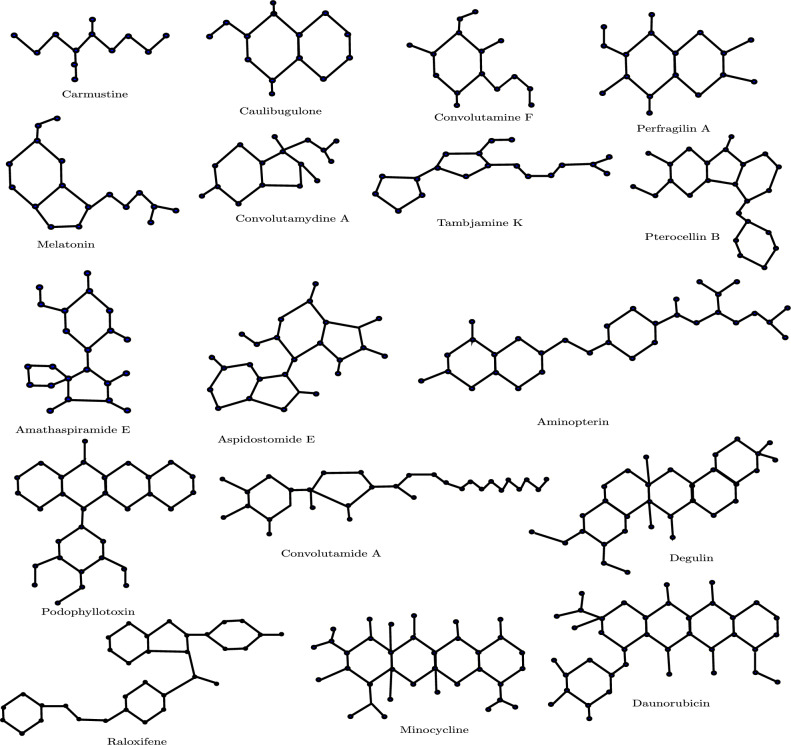
Molecular Structures of some anticancer drugs.

In this study, we employ the Euler–Sombor index to model four important physico-chemical properties—boiling point (BP), melting point (MP), enthalpy (E), and molar refraction (MR) of 17 selected anticancer drugs. The experimental values of these properties are obtained from ChemSpider and are also reported in^7,28^. The molecular structures corresponding to the anticancer drugs namely Carmustine, Caulibugulone E, Convolutamine F, Perfragilin A, Melatonin, Convolutamydine A, Tambjamine K, Pterocellin B, Amathaspiramide E, Aspidostomide E, Aminopterin, Podophyllotoxin, Convolutamide A, Deguelin, Minocycline, Daunorubicin, and Raloxifene, are shown in Fig. 1. The experimental data for these compounds are summarized in Table 1.

**Table 1:** Some anticancer drugs with its physico-chemical property value.

Drug	BP ($^\circ$C)	MP ($^\circ$C)	E (kJ/mol)	MR (cm$^3$)
Carmustine	309.6	120.99	63.8	46.6
Caulibugulone E	373	129.46	62	52.2
Convolutamine F	387.7	128.67	63.7	73.8
Perfragilin A	431.5	187.62	68.7	63.6
Melatonin	512.8	182.51	78.4	67.6
Convolutamydine A	504.9	199.2	81.6	68.2
Trambjamine K	391.7	-	64.1	76.6
Pterocellin B	521.6	199.88	79.5	87.4
Amathaspiramide E	572.7	209.72	90.3	89.4
Aspidostomide E	798.8	-	116.2	116
Aminopterin	782.27	344.45	-	114
Podophyllotoxin	597.9	235.86	93.6	104.3
Convolutamide A	629.9	-	97.9	130.1
Deguelin	560.1	213.39	84.3	105.1
Minocycline	803.3	326.3	122.5	116
Daunorubicin	770	208.5	117.6	130
Raloxifene	728.2	289.58	110.1	136.6

**Table 2:** Anticancer drugs with their calculated Sombor-type topological indices.

Drug	EuSO	SO	ESO	HSO
Carmustine	40.208	33.4151	146.254	20.9357
Caulibugulone	62.8283	52.0442	257.9097	27.474
Convolutamine F	61.2566	51.0086	247.9256	29.6554
Perfragilin A	70.1158	58.5535	285.3165	35.009
Melatonin	73.3357	60.8096	290.4228	33.2709
Convolutamydine A	75.6344	63.5543	331.0086	37.7895
Tambjamine K	80.2639	66.4664	313.0502	36.0993
Pterocelin B	114.9624	94.8907	476.3849	46.7759
Amathaspiramide E	106.7858	88.9606	470.4344	47.5947
Aspidostomide E	129.3271	107.3998	561.6365	55.8195
Aminopterin	143.8009	120.1857	592.4139	69.3937
Podophyllotoxin	142.7442	117.6939	603.0033	56.7111
Convolutamide A	131.2203	109.1877	533.1565	61.184
Deguelin	161.3631	134.88	734.123	72.1799
Raloxifene	153.1637	126.2388	613.5556	62.039
Minocycline	179.4372	150.7844	837.6072	85.7366
Daunorubicin	189.1624	157.721	832.602	84.8306

Here we consider three types of models, namely

1. $$Y=AX+B$$ - linear model
2. $$Y=A+BX+CX^2$$ - quadratic model
3. $$Y=A\ln (X)+B$$ - logarithmic model.

In the above models, *Y* is the variable that depends on the independent variable *X*, while the regression constants are represented by *A*, *B* and *C*. We denote the correlation coefficient, standard error of the model, the *F*-test value and the significance by *r*, *SE*, *F* and *SF* respectively.

**Table 3:** Coefficient of correlation between EuSo and Sombor-type topological indices.

Models	SO	ESO	HSO
Linear	0.9998	0.993	0.9567
Quadratic	0.9998	0.9939	0.9616
Logarithmic	0.9799	0.9683	0.9258

Table 3 presents the correlation coefficients between the EuSO index and different Sombor-type topological indices considered in Table 2. It is observed that the EuSO index shows an excellent correlation with the SO index across all three models, with $r \approx 0.9998$. Strong correlations are also observed with ESO and HSO indices, particularly under quadratic and linear models, while the logarithmic model gives slightly weaker associations.

### Boiling point v/s Euler–Sombor index

Based on data presented in Tables 1 and 2, we get follwing plots for EuSO and BP.

**Fig. 2:**
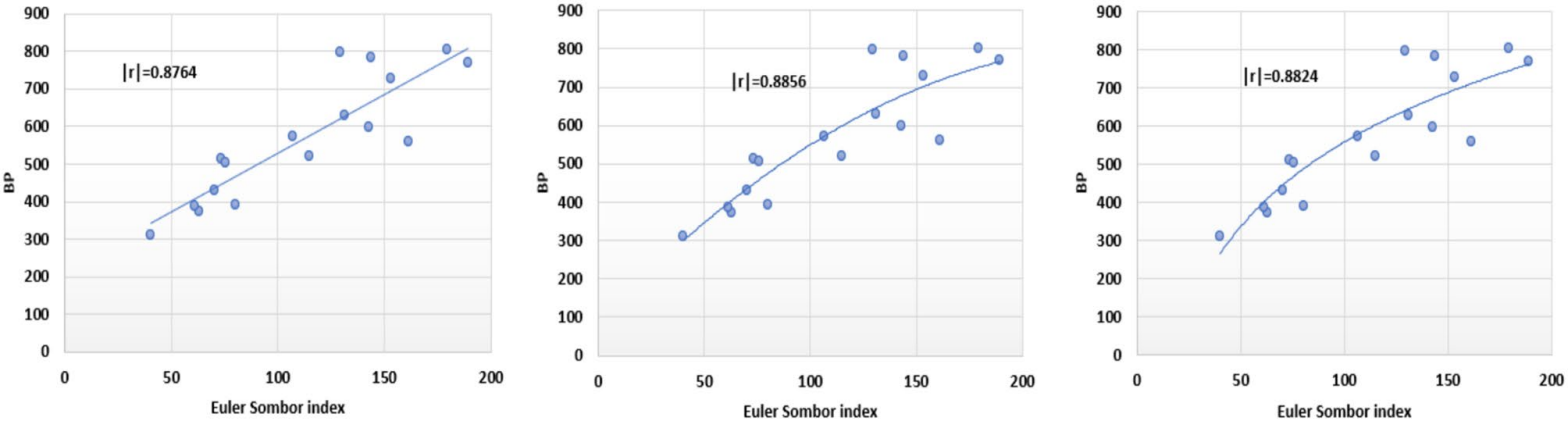
Linear, quadratic and logarithmic scatter plot of EuSo v/s BP.

The following models are depicted in Fig. 2.

**Fig. 3:**
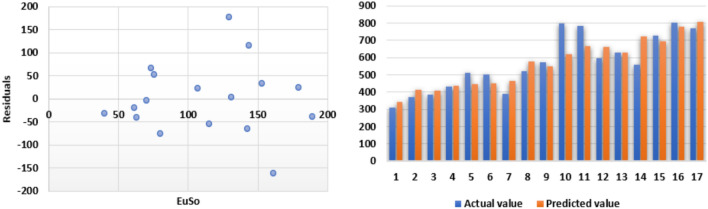
Residual plot of linear model and experimental v/s predicted BP.

**Fig. 4:**
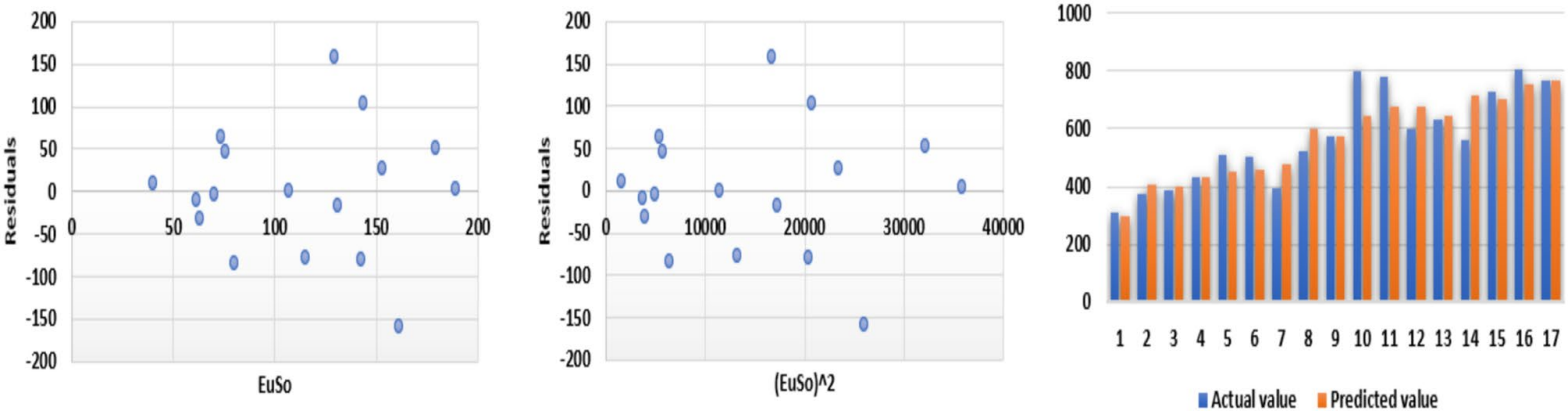
Residual plot of quadratic model and experimental v/s predicted BP.

**Fig. 5:**
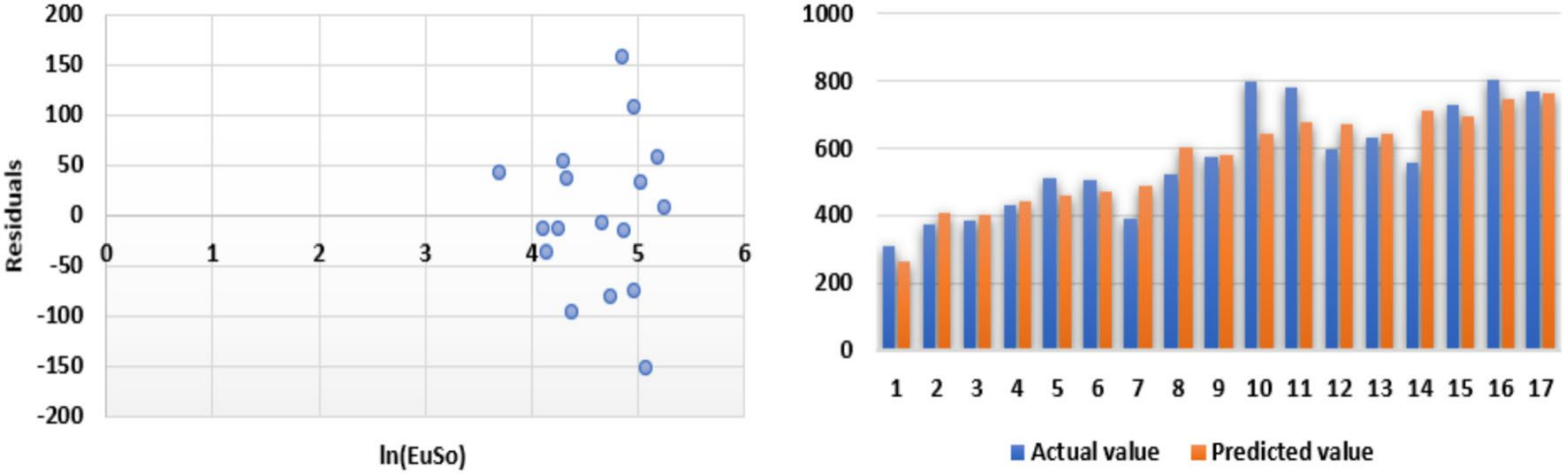
Residual plot of logarithmic model and experimental v/s predicted BP.

- Linear model (Fig. 3): $BP=3.1332(EuSO)+216.12$, $r^2=0.768$.
- Quadratic model (Fig. 4): $BP=-0.0118(EuSO)^2+5.8375(EuSO)+84.0116$, $r^2=0.7842$.
- Logarithmic model (Fig. 5): $BP=320.51\ln (EuSO)-917,2$, $r^2=0.7786$.

**Table 4:** Statistical analysis for the EuSO index.

Models	r	SE	F	SF
Linear model	0.8764	80.79502	49.64629	$3.96\times 10^{-6}$
Quadratic model	0.8856	80.66109	25.43057	$2.18\times 10^{-5}$
Logarithmic model	0.8824	78.9307	52.7362	$2.78\times 10^{-6}$

**Table 5:** Comparing actual and predicted values of BP using linear, quadratic and logarithmic models.

BP				
Drug	Actual values	Linear model	Quadratic model	Logarithmic model
		Predicted value	Predicted value	Predicted value
Carmustine	309.6	342.0972	299.6660	266.7893
Caulibugulone	373.0	412.9708	404.2282	409.8463
Convolutamine F	387.7	408.0464	397.3531	401.7265
Perfragilin A	431.5	435.8039	435.3445	445.0201
Melatonin	512.8	445.8925	448.6940	459.4109
Convolutamydine A	504.9	453.0947	458.0746	469.3030
Tambjamine K	391.7	467.5998	476.5887	488.3442
Pterocelin B	521.6	576.3167	599.2614	603.4993
Amathaspiramide E	572.7	550.6979	572.9111	579.8519
Aspidostomide E	798.8	621.3240	641.7355	641.2363
Aminopterin	782.27	666.6731	679.6101	675.2377
Podophyllotoxin	597.9	663.3623	677.0122	672.8738
Convolutamide A	629.9	627.2558	646.9704	645.8942
Deguelin	560.1	721.6988	718.9319	712.1695
Raloxifene	728.2	696.0086	701.4787	695.4548
Minocycline	803.3	778.3284	751.8047	746.1976
Daunorubicin	770.0	808.7992	766.3047	763.1144

### Melting point v/s Euler–Sombor index

**Fig. 6:**
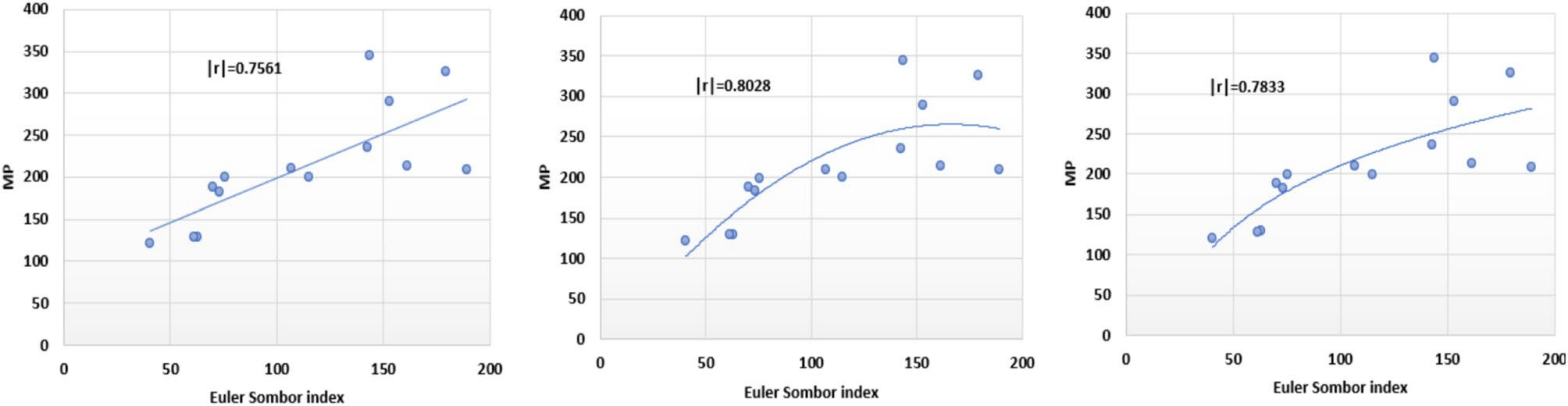
Linear, quadratic and logarithmic scatter plot of EuSo v/s MP.

The following models are depicted in Fig. 6.

**Fig. 7:**
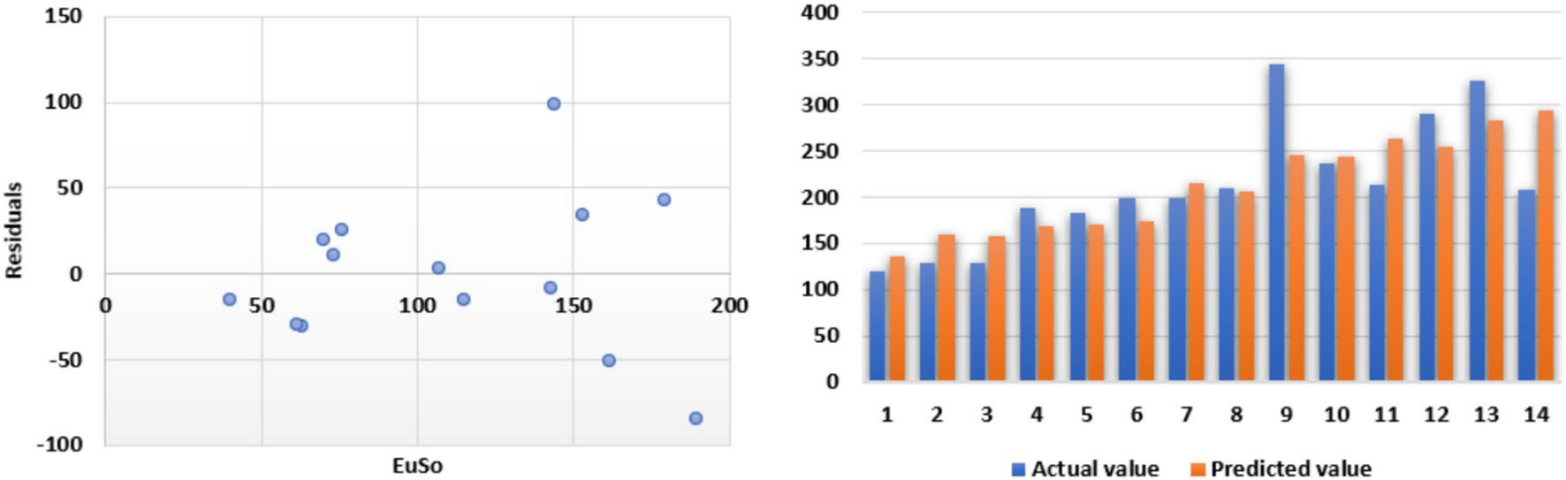
Residual plot of linear model and experimental v/s predicted MP.

**Fig. 8:**
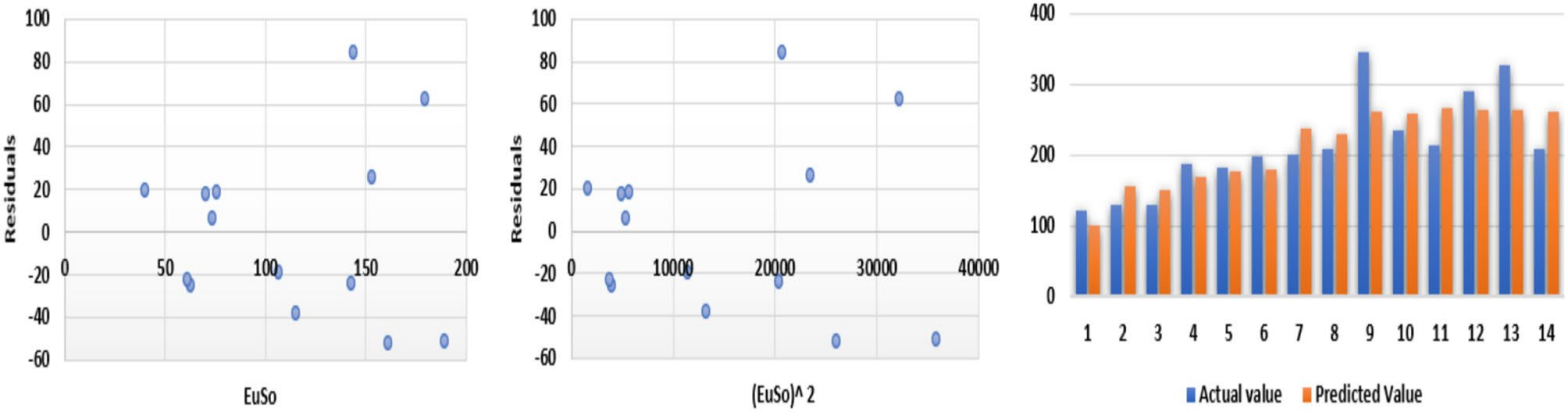
Residual plot of quadratic model and experimental v/s predicted MP.

**Fig. 9:**
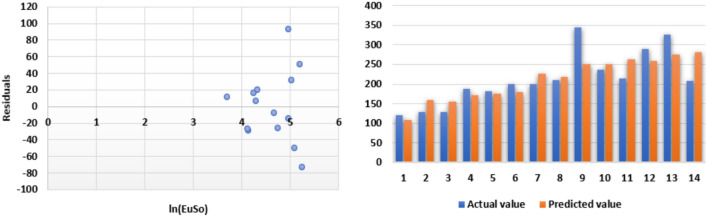
Residual plot of logarithmic model and experimental v/s predicted MP.

- Linear model (Fig. 7): $MP=1.055(EuSO)+93.905$, $r^2=0.5717$.
- Quadratic model (Fig. 8): $MP=-0.0104(EuSO)^2+3.4411(EuSO)-20.326$, $r^2=0.6445$.
- Logarithmic model (Fig. 9): $MP=111.28\ln (EuSO)-301.78$, $r^2=0.6135$.

**Table 6:** Statistical analysis for the EuSO index.

Models	r	SE	F	SF
Linear model	0.7561	46.67204	16.01498	0.001756
Quadratic model	0.8028	44.41007	9.970731	0.003386
Logarithmic model	0.7833	44.33241	19.04996	0.000921

**Table 7:** Comparing actual and predicted values of MP using linear, quadratic and logarithmic models.

MP				
Drug	Actual values	Linear model	Quadratic model	Logarithmic model
		Predicted value	Predicted value	Predicted value
Carmustine	120.99	136.3255	101.2965	109.2817
Caulibugulone	129.46	160.1906	155.0062	158.9492
Convolutamine F	128.67	158.5324	151.6168	156.1301
Perfragilin A	187.62	167.8792	170.0533	171.1610
Melatonin	182.51	171.2763	176.3514	176.1573
Convolutamydine A	199.20	173.7015	180.7163	179.5917
Pterocelin B	199.88	215.1938	238.4470	226.1829
Amathaspiramide E	209.72	206.5672	229.0812	217.9728
Aminopterin	344.45	245.6194	260.4286	251.0895
Podophyllotoxin	235.86	244.5045	259.9270	250.2687
Deguelin	213.39	264.1481	265.3787	263.9116
Raloxifene	289.58	255.4974	263.8624	258.1086
Minocycline	326.30	283.2168	263.8051	275.7258
Daunorubicin	208.50	293.4772	260.1596	281.5990

### Enthalpy v/s Euler–Sombor index

**Fig. 10:**
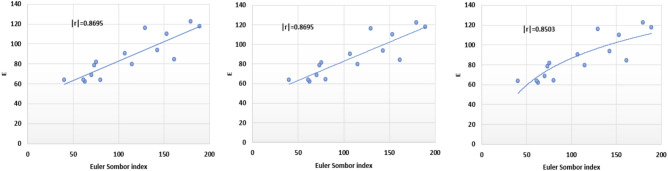
Linear, quadratic and logarithmic scatter plot of EuSo v/s E.

The following models are depicted in Fig. 10.

**Fig. 11:**
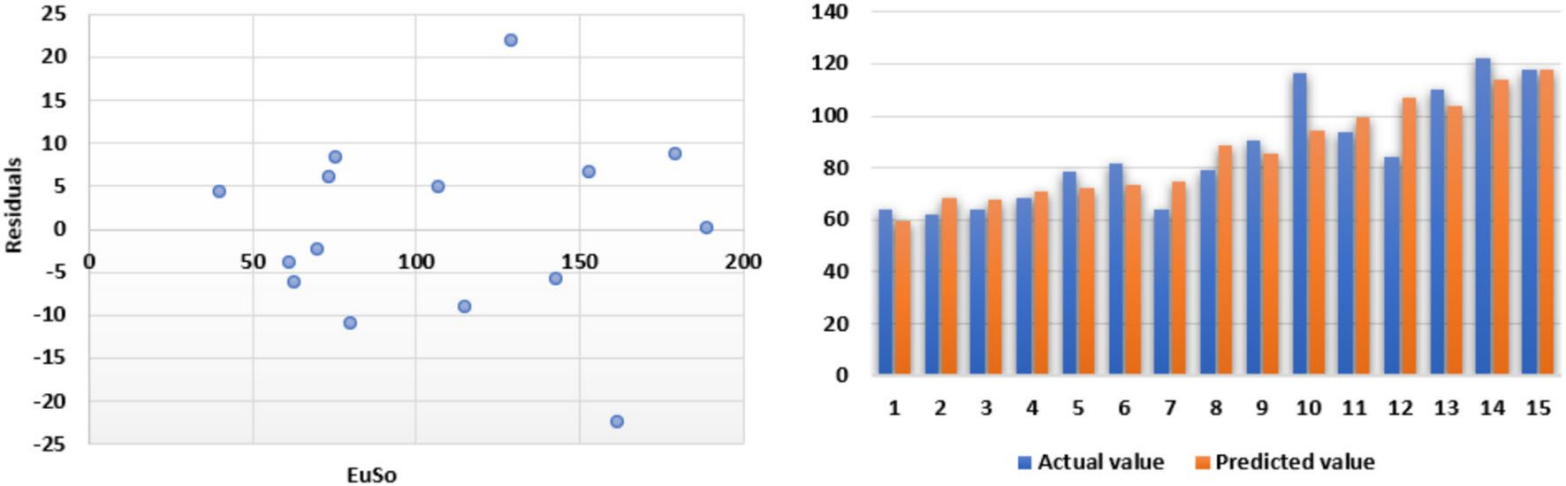
Residual plot of linear model and experimental v/s predicted E.

**Fig. 12:**
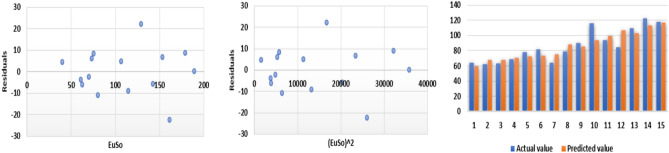
Residual plot of quadratic model and experimental v/s predicted E.

**Fig. 13:**
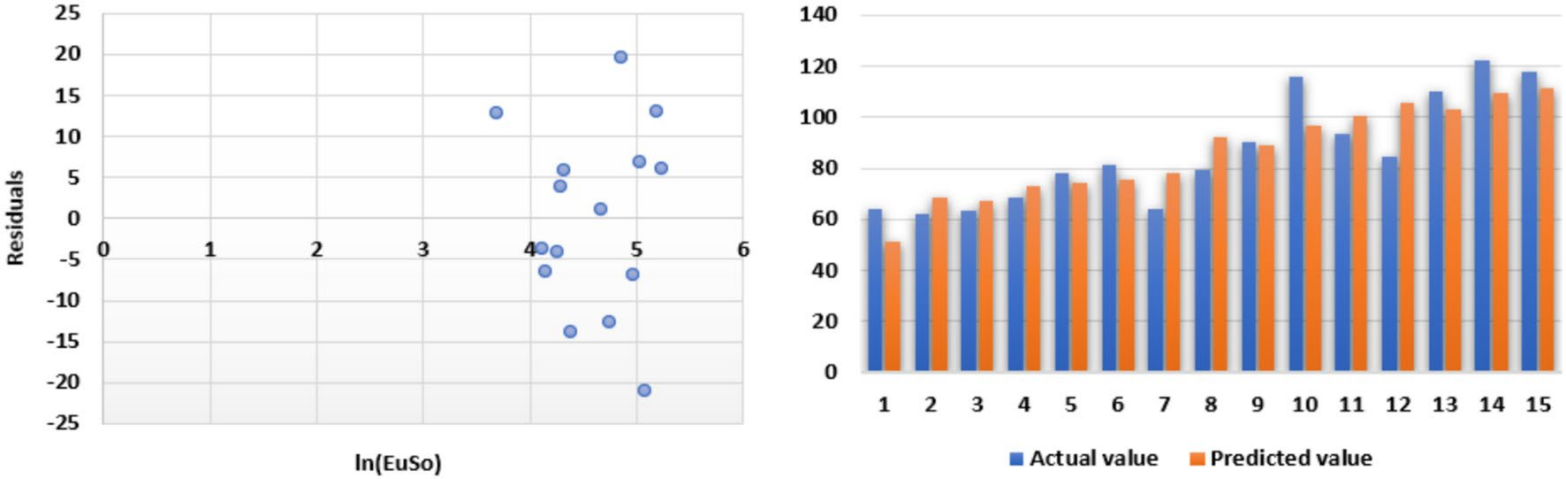
Residual plot of logarithmic model and experimental v/s predicted E.

- Linear model (Fig. 11): $E=0.3906(EuSO)+43.703$, $r^2=0.7561$.
- Quadratic model (Fig. 12):$E=-1\times 10^{-5}(EuSO)+0.3935(EuSO)+43.564$, $r^2=0.7561$.
- Logarithmic model (Fig. 13): $E=39.099\ln (EuSO)-93.411$, $r^2=0.723$.

**Table 8:** Statistical analysis for the EuSO index.

Models	r	SE	F	SF
Linear model	0.8695	10.92767	40.29748	$2.54\times 10^{-5}$
Quadratic model	0.8695	11.37386	18.59894	0.000211
Logarithmic model	0.8503	11.6462	33.92383	$5.93\times 10^{-5}$

**Table 9:** Comparing actual and predicted values of E using linear, quadratic and logarithmic models.

E				
Drug	Actual values	Linear model	Quadratic model	Logarithmic model
		Predicted value	Predicted value	Predicted value
Carmustine	63.8	59.4093	59.3653	51.0250
Caulibugulone	62.0	68.2453	68.2371	68.4766
Convolutamine F	63.7	67.6314	67.6211	67.4861
Perfragilin A	68.7	71.0920	71.0926	72.7675
Melatonin	78.4	72.3498	72.3539	74.5230
Convolutamydine A	81.6	73.2477	73.2542	75.7298
Trambjamine K	64.1	75.0561	75.0669	78.0526
Pterocelin B	79.5	88.6102	88.6365	92.1005
Amathaspiramide E	90.3	85.4162	85.4416	89.2157
Aspidostomide E	116.2	94.2214	94.2455	96.7040
Podophyllotoxin	93.6	99.4625	99.4799	100.5635
Deguelin	84.3	106.7355	106.7363	105.3572
Raloxifene	110.1	103.5326	103.5418	103.3182
Minocycline	122.5	113.7956	113.7723	109.5083
Daunorubicin	117.6	117.5946	117.5548	111.5720

### Molar refraction v/s Euler–Sombor index

**Fig. 14:**
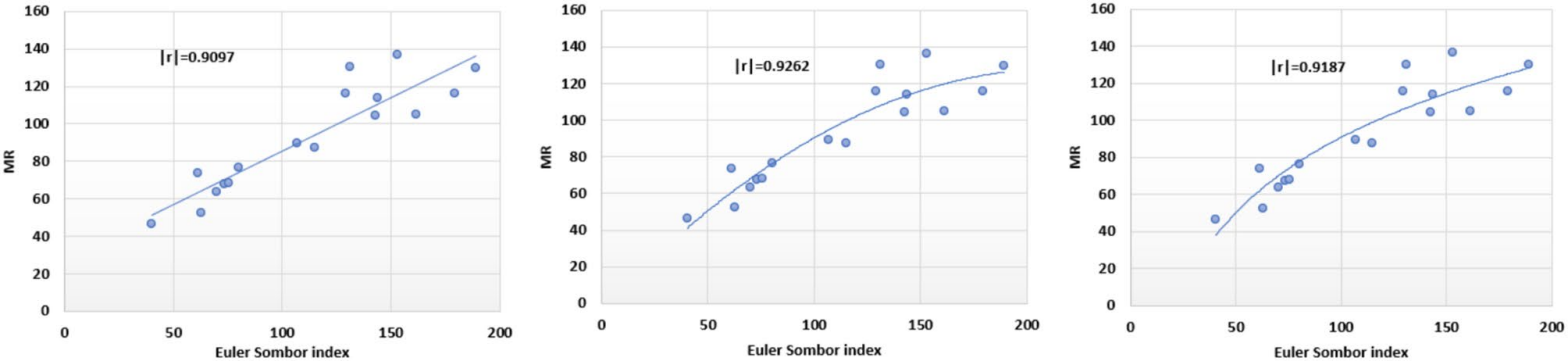
Linear, quadratic and logarithmic scatter plot of EuSo v/s MR.

The following models are depicted in Fig. 14.

**Fig. 15:**
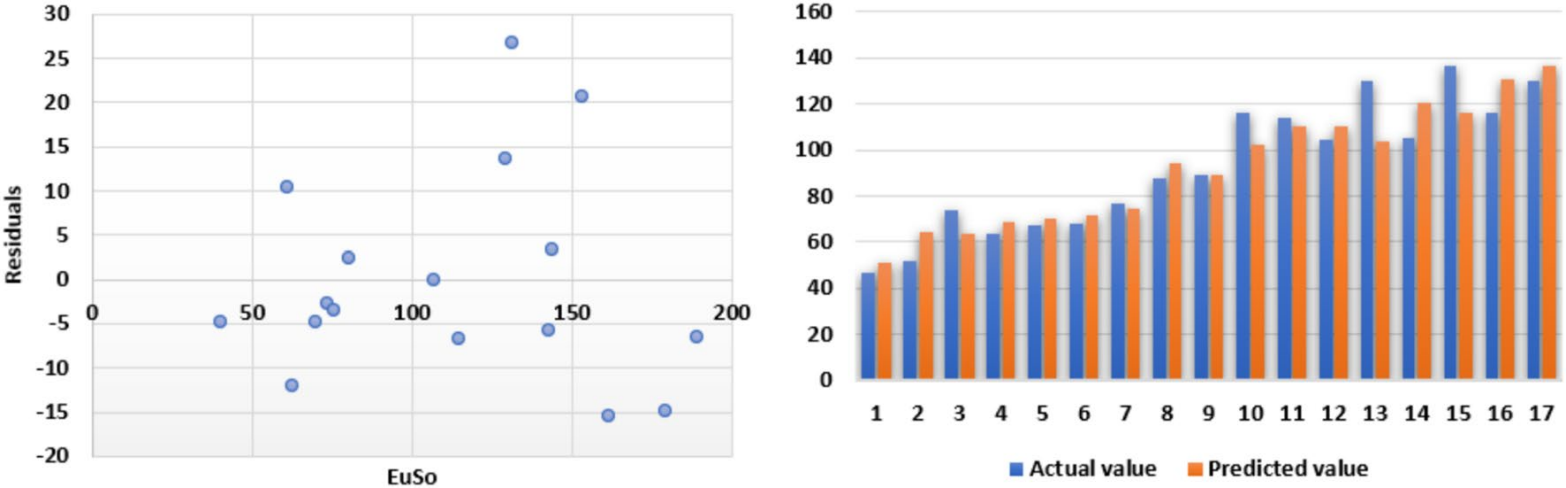
Residual plot of linear model and experimental v/s predicted MR.

**Fig. 16:**
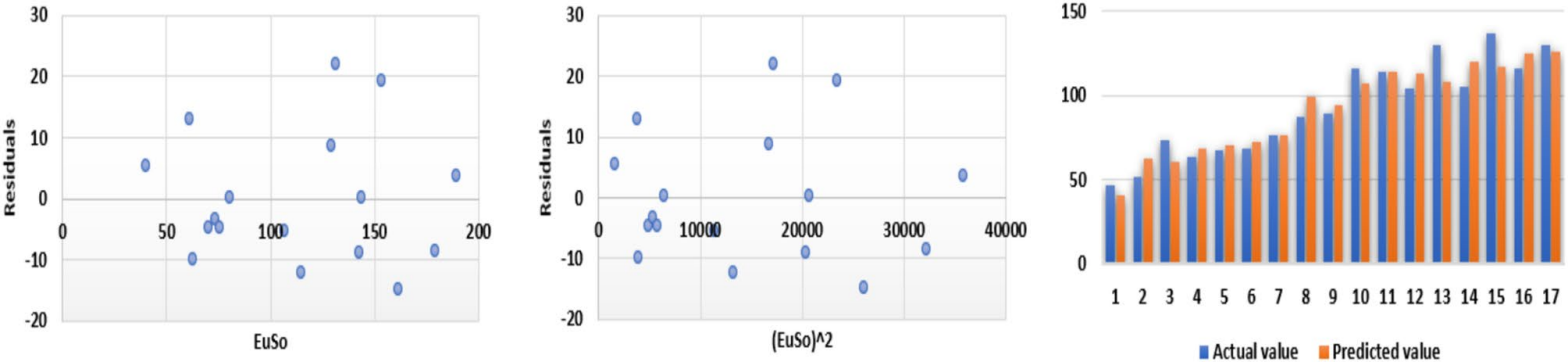
Residual plot of quadratic model and experimental v/s predicted MR.

**Fig. 17:**
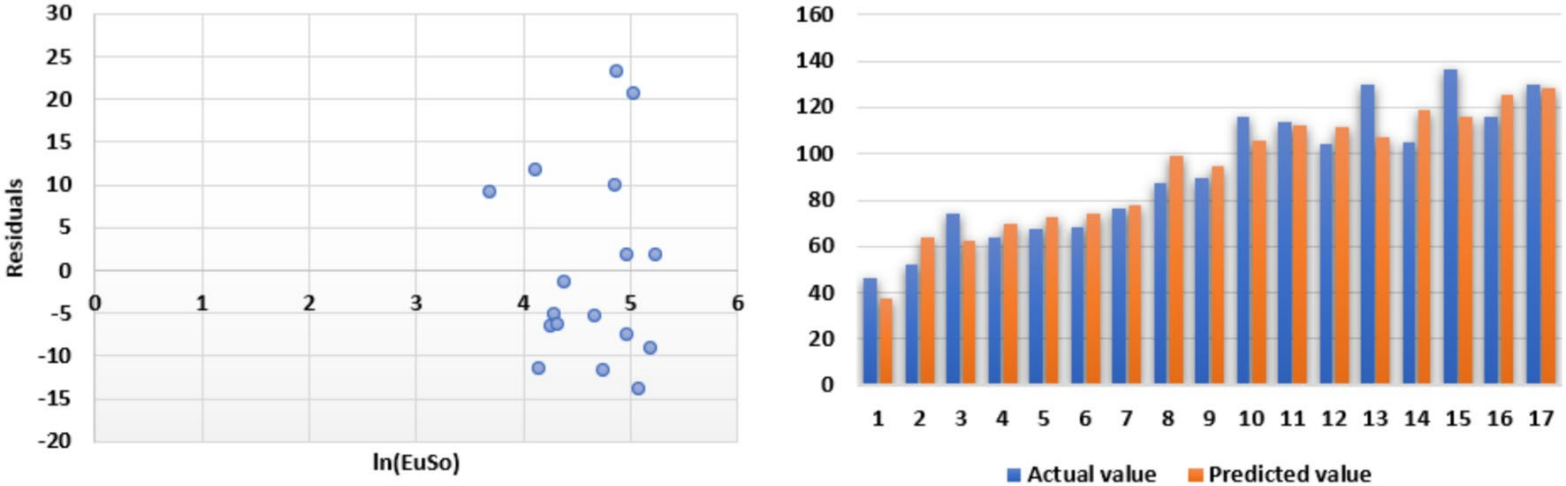
Residual plot of logarithmic model and experimental v/s predicted E.

- Linear model (Fig. 15): $MR=0.5718(EuSO)+28.361$, $r^2=0.8276$.
- Quadratic model (Fig. 16): $MR=-0.0028(EuSO)^2+1.2224(EuSO)-3.4189$, $r^2=0.8579$.
- Logarithmic model (Fig. 17): $MR=58.671\ln (EuSO)-179.29$, $r^2=0.8441$.

**Table 10:** Statistical analysis for the EuSO index.

Models	r	SE	F	SF
Linear model	0.9097	12.24446	71.99466	$4.14\times 10^{-7}$
Quadratic model	0.9262	11.50645	42.25601	$1.17\times 10^{-6}$
Logarithmic model	0.9187	11.64332	81.20948	$1.93\times 10^{-7}$

**Table 11:** Comparing actual and predicted values of MR using linear, quadratic and logarithmic models.

MR				
Drug	Actual values	Linear model	Quadratic model	Logarithmic model
		Predicted value	Predicted value	Predicted value
Carmustine	46.6	51.3526	41.1447	37.4412
Caulibugulone	52.2	64.2870	62.1837	63.6284
Convolutamine F	73.8	63.3883	60.8158	62.1420
Perfragilin A	63.6	68.4540	68.3435	70.0671
Melatonin	67.6	70.2952	70.9691	72.7014
Convolutamydine A	68.2	71.6096	72.8076	74.5122
Trambjamine K	76.6	74.2567	76.4192	77.9978
Pterocelin B	87.4	94.0975	99.6174	99.0774
Amathaspiramide E	89.4	89.4221	94.7660	94.7486
Aspidostomide E	116.0	102.3113	107.2218	105.9853
Aminopterin	114.0	110.5875	113.6998	112.2094
Podophyllotoxin	104.3	109.9833	113.2671	111.7767
Convolutamide A	130.1	103.3939	108.1367	106.8380
Deguelin	105.1	120.6297	119.9641	118.9699
Raloxifene	136.6	115.9412	117.2572	115.9102
Minocycline	116.0	130.9646	124.5837	125.1989
Daunorubicin	130.0	136.5255	126.3024	128.2956

###  Results and discussions

The regression analysis of the EuSO index with respect to four physico-chemical properties—boiling point, melting point, enthalpy, and molar refraction was carried out using linear, quadratic, and logarithmic models. The Figs. 3, 4, 5, 7, 8, 9, 11, 12, 13, 15, 16 and 17 consist of residual plots and in particular bar diagrams are used to compare the experimental properties with predicted properties. In bar diagrams blue bars indicate the actual values, whereas orange bars represent the predicted values. Tables 5, 7, 9 and 11 present comparisons of actual and predicted values for the predictive models within the linear, quadratic, and logarithmic regression frameworks. This visualization facilitates a direct comparison between actual and predicted data. From Tables 4, 6, 8 and 10 we can observe the following results.

#### Coefficient of correlation

- For boiling point and enthalpy, all models give strong correlations ($r \approx 0.85-0.88$), showing a good linear association between the EuSO index and the property.
- For melting point, *r* values are slightly lower ($\approx 0.75-0.80$), indicating weaker predictive strength.
- For molar refraction, very high correlations are obtained ($r>0.90$), confirming that the EuSO index strongly predict this property across models.

#### Standard error

- SE values for boiling point are all large ($\approx 80$), suggesting higher variability and less precise predictions compared to the other properties.
- For melting point, SE decreases to ($\approx 44$–46), indicating slightly better precision.
- For enthalpy, SE is much smaller ($\approx 11$), showing high accuracy of model.
- For molar refraction, SE values are again small ($\approx 11$–12), reinforcing very precise prediction.

#### *F*-value and *SF*-value

- All three models showed strong correlation ($r \approx 0.87$) for boiling point; however, the logarithmic model displayed the highest *F*-value (52.7362) and the lowest *SF*-value ($2.78 \times 10^{-6}$), indicating superior statistical significance and predictive accuracy compared to the linear and quadratic models.
- A similar trend was observed in case of melting point, where the logarithmic model again outperformed the other models with a higher *F*-value (19.05) and smaller *SF* (0.000921), suggesting that it provides a more reliable prediction, while the quadratic model was the least effective.
- Although all models had strong correlation ($r \approx 0.85$) for enthalpy, the linear model gave the best performance with the highest *F*-value (40.29748) and the smallest *SF*-value ($2.54 \times 10^{-5}$), making it the most accurate for this property.
- A very high correlations were observed for all models ($r> 0.90$) in case of molar refraction, but the logarithmic model again proved superior, yielding the highest *F*-value (81.20948) and the lowest *SF*-value ($1.93 \times 10^{-7}$), despite the quadratic model having a slightly lower SE.

Overall, the analysis reveals that the logarithmic regression model provides the most reliable predictions for boiling point, melting point, and molar refraction, while the linear regression model is slightly better suited for enthalpy. The quadratic model, in contrast, consistently showed weaker performance across all properties, with lower statistical significance and less predictive accuracy. These results highlight the strong applicability of logarithmic regression in modeling the relationship between the EuSO index and various physico-chemical properties, except in the case of enthalpy, where a linear relationship appears more appropriate.

### Techniques used for computation of results

The values of the Euler–Sombor (EuSO) index were computed using MATLAB, and these indices were then correlated with the selected properties through QSPR analysis. Three different regression models—linear, quadratic, and logarithmic—were developed in Microsoft Excel to establish predictive relationships. Statistical parameters including correlation coefficient (*r*), standard error (SE), F-value, and standard error of model (SF) were determined to assess the significance and accuracy of the models. To further validate the analysis, scatter plots, residual plots, and bar diagrams were constructed to compare actual and predicted values, providing a comprehensive evaluation of the predictive performance of the EuSO index. The workflow of the QSPR methodology illustrating data collection, computation of Sombor index variants, statistical modeling, and correlation analysis for anti-cancer drug molecules depicted in Fig. 18.

### Methodology

**Fig. 18:**
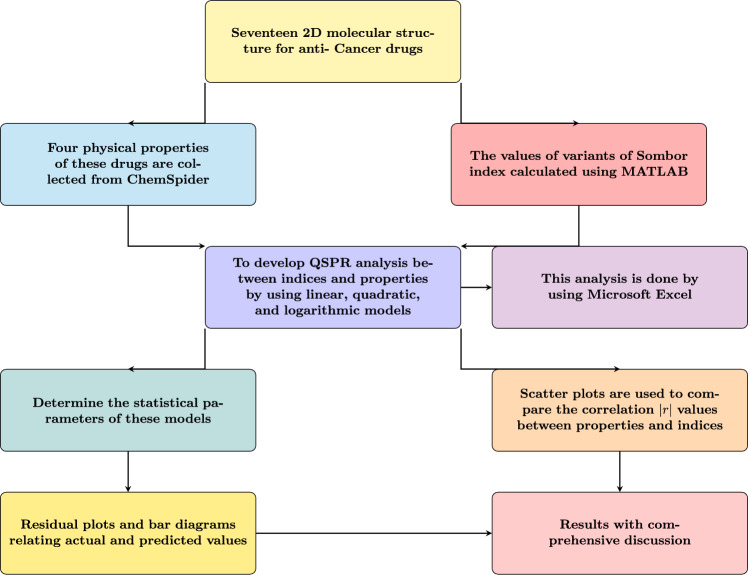
Workflow of the QSPR methodology for selected anticancer drug molecules.

### Limitations and future work

Although the Euler–Sombor index shows strong correlations with the physicochemical properties of the selected anticancer drugs, some limitations should be noted. Despite the presence of strong correlations, the statistical evaluation indicates that the boiling point (BP) prediction model exhibits a comparatively high standard error (SE $\approx 80$). This reflects lower predictive precision for BP when compared with the enthalpy and molar refraction models, which demonstrate substantially smaller SE values ($\approx 10$–12). Moreover, the analysis is based on a limited number of drug molecules, which may affect the general applicability of the results; however, the main aim of this work was to study structure–property relationships rather than to build highly general predictive models. In future studies, larger datasets, additional molecular descriptors, and improved modeling techniques may be considered to enhance prediction accuracy.

## Unicyclic graph with third minimum Euler–Sombor index

The Euler–Sombor index of a cycle $C_{n}$ is $2\sqrt{3}n$, and it is the minimum among all unicyclic graphs. In this section, we identify unicyclic graphs that have a specified girth and possess the third minimum Euler–Sombor index. Our study, indeed extends the following result obtained by Su and Tang.

### Theorem 3.1

^1^ Let $G\in \mathcal {U}_{n,k}$ $(3\le k\le n-2)$. Then $EuSO(G)\ge 3\sqrt{19}+\sqrt{7}+2(n-4)\sqrt{3}$ equality holds if and only if $G\cong U_{n,k}$.

**Fig. 19:**
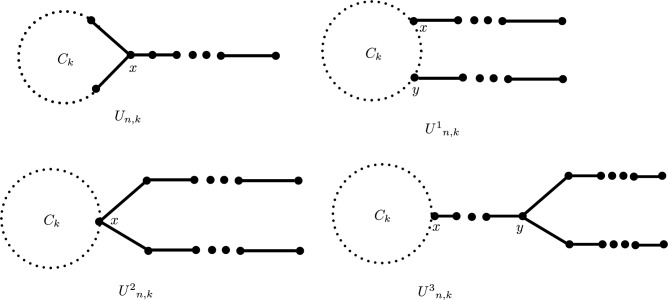
Graphs $$U_{n,k}$$, $$U^1_{n,k}$$, $$U^2_{n,k}$$ $$(3\le k\le n-2)$$ and $$U^3_{n,k}$$ $$(3\le k\le n-3)$$.

We need the following classes of unicyclic graphs (see Fig. 19).

Class $U^{1}_{n,k}$: This class consists of unicyclic graphs of order *n* formed by attaching a pendant vertex of a path of length *s* to a vertex *x* in the cycle $C_{k}$, and then a pendant vertex of another path of length *t* is attached to some other vertex *y* ($x\ne y$) in the same cycle $C_{k}$, where $s\ge t\ge 1$.

Class $U^{2}_{n,k}$: A unicyclic graph of order *n* in this class can be obtained by attaching a pendant vertex in each of the two paths of length *s* and *t* to a vertex *x* of the cycle $C_k$, where $s\ge t\ge 1$.

Class $U^{3}_{n,k}$: Consists of graphs obtained by attaching a path of length *t* between a vertex *x* of the cycle $C_k$ and a non-pendant vertex *y* of a path of length *s*, where $s\ge 2, t\ge 1$. The following two lemmas proved in^1^ are important to prove our results.

### Lemma 3.2

^1^ Let *G* be a graph and let $P:x_0-x_1-x_2-\cdots x_{k-1}-x_{k}-\cdots -x_p$ be a path in *G* with $d_G(x_0)=d_G(x_p)=1$, $d_G(x_i)=2$ for $2\le i\le p-1$ and $i\ne k$, and $d_G(x_k)\ge 3$. Then for the graph $G'=G-x_kx_{k-1}+x_{k-1}x_p$, $EuSO(G)> {EuSO}(G')$.

### Lemma 3.3

^1^ Let $P_{1}:x-x_1-x_2-\ldots -x_p$ and $P_{2}:y-y_1-y_2-\dots -y_q$, be two non intersecting paths in a graph $\Gamma$ such that $d_{\Gamma }(x_i)=2$ $(1\le i\le p-1)$, $d_{\Gamma }(y_j)=2$ $(1\le j\le q-1)$, $d_{\Gamma }(x)\ge 3$, $d_{\Gamma }(y)\ge 3$ and $d_{\Gamma }(x_{p})=d_{\Gamma }(y_{q})=1$. Then for the graph $\Gamma ^{\prime }=\Gamma -xx_1+x_1y_q$, $EuSO(\Gamma )>EuSO(\Gamma ^{\prime })$.

### Lemma 3.4

Let *G* be a graph that belongs to one of the classes $U^1_{n,k}$ or $U^2_{n,k}$, where $3\le k\le n-4$. Then $EuSO(G)\ge 4\sqrt{19}+2\sqrt{7}+(2n-11)\sqrt{3}$. Equality holds if and only if $G\in U^1_{n,k}$ with $s\ge t>1$ and $x\sim y$.

### Proof

We have the following cases. Case 1. Suppose $G\in U^1_{n,k}$. If $x\sim y$ in *G*, then

$$\begin{aligned} EuSO(G) = {\left\{ \begin{array}{ll} 3\sqrt{19}+\sqrt{7}+\sqrt{13}+(2n-9)\sqrt{3}, & t=1,\, s>t \\ \\ 4\sqrt{19}+2\sqrt{7}+(2n-11)\sqrt{3} , & s\ge t>1. \end{array}\right. } \end{aligned}$$

Otherwise, $x\not \sim y$ in *G*. In this case,

$$\begin{aligned} EuSO(G) = {\left\{ \begin{array}{ll} 5\sqrt{19}+\sqrt{7}+\sqrt{13}+2(n-7)\sqrt{3}, & t=1,\, s>t \\ \\ 6\sqrt{19}+2\sqrt{7}+2(n-8)\sqrt{3} , & s\ge t>1. \end{array}\right. }\quad \quad \square \end{aligned}$$

Case 2. Suppose $G\in U^2_{n,k}$. Then

$$\begin{aligned} EuSO(G) = {\left\{ \begin{array}{ll} \sqrt{21}+7\sqrt{7}+2(n-5)\sqrt{3}, & t=1,\, s>t \\ \\ 10\sqrt{7}+2(n-6)\sqrt{3}, & s\ge t>1. \end{array}\right. } \end{aligned}$$

Now, $4\sqrt{19}+2\sqrt{7}+(2n-11)\sqrt{3}$ < $6\sqrt{19}+2\sqrt{7}+2(n-8)\sqrt{3}$ < $3\sqrt{19}+\sqrt{7}+\sqrt{13}+(2n-9)\sqrt{3}$ < $5\sqrt{19}+\sqrt{7}+\sqrt{13}+2(n-7)\sqrt{3}$ < $10\sqrt{7}+2(n-6)\sqrt{3}$ < $\sqrt{21}+7\sqrt{7}+2(n-5)\sqrt{3}$. Thus, we arrive at the result.

### Lemma 3.5

Let *G* be a graph in $U^3_{n,k}$ ($3\le k\le n-4$). Then $EuSO(G)\ge 4\sqrt{19}+2\sqrt{7}+(2n-11)\sqrt{3}$. Equality holds if and only if $x\sim y$ in *G* (i.e., $t=1$) and *y* is not a quasi-pendant vertex.

### Proof

Assume that $t=1$. Since $k\le n-4$, $s\ge 3$. So, *y* is adjacent to at most one pendant vertex. Therefore,

$$\begin{aligned} EuSO(G) = {\left\{ \begin{array}{ll} 3\sqrt{19}+\sqrt{13}+\sqrt{7}+(2n-9)\sqrt{3},& \textit{if}\,\, y\,\, \text {is a quasi-pendant vertex} \\ \\ 4\sqrt{19}+2\sqrt{7}+(2n-11)\sqrt{3}, & \text {if}\,\, y\,\, \text {is not a quasi-pendant vertex}. \end{array}\right. } \end{aligned}$$

Suppose $$t>1$$. Then

$$\begin{aligned} EuSO(G) = {\left\{ \begin{array}{ll} 4\sqrt{19}+2\sqrt{13}+2(n-6)\sqrt{3},& \text {if}\,\, y\,\, \text {is adjacent to two pendant vertices} \\ \\ 5\sqrt{19}+\sqrt{13}+\sqrt{7}+2(n-7)\sqrt{3},& \text {if} \,\,y\,\, \text {is adjacent to one pendant vertex}\\ \\ 6\sqrt{19}+2\sqrt{7}+2(n-8)\sqrt{3}, & \text {if}\,\, y\,\, \text {is not adjacent to any pendant vertex.} \end{array}\right. } \end{aligned}$$

Now, $4\sqrt{19}+2\sqrt{7}+(2n-11)\sqrt{3}$ < $6\sqrt{19}+2\sqrt{7}+2(n-8)\sqrt{3}$ < $3\sqrt{19}+\sqrt{13}+\sqrt{7}+(2n-9)\sqrt{3}$ < $5\sqrt{19}+\sqrt{13}+\sqrt{7}+2(n-7)\sqrt{3}$ < $4\sqrt{19}+2\sqrt{13}+2(n-6)\sqrt{3}$, and hence the result follows. $\square$

**Fig. 20:**
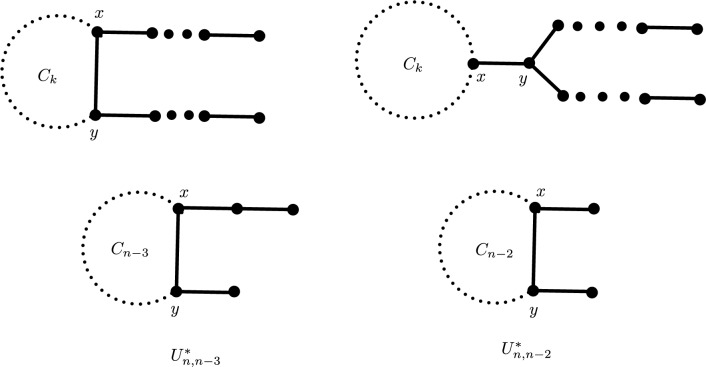
The graphs $U^*_{n,k}$ $(3\le k\le n-4)$, $U^*_{n,n-3}$ and $U^*_{n,n-2}.$.

Let $U^*_{n,k}$ ($3\le k\le n-4$) denote the class of unicyclic graphs consisting of graphs that belong to $U^1_{n,k}$ with $s\ge t\ge 2$ and $x\sim y$, and also the graphs in $U^2_{n,k}$ with $x\sim y$ (i.e., $t=1$) and *y* is not a quasi-pendant vertex, see Fig. 20. Note that the Euler–Sombor index of a graph in $U^*_{n,k}$ is equal to $4\sqrt{19}+2\sqrt{7}+(2n-11)\sqrt{3}$, see Lemmas 3.4 and 3.5. Let $U^{*}_{n,n-3}$ and $U^{*}_{n,n-2}$ be unicyclic graphs of order n as shown in Fig. 20.

### Proposition 3.6

Let $$G\in \mathcal {U}_{n,k}$$ and $$G\not \cong U_{n,k}$$.

(i) For $k=n-3$, $EuSO(G)\ge 3\sqrt{19}+2\sqrt{6}+\sqrt{13}+\sqrt{7}+2(n-6)\sqrt{3}$. Equality holds if and only if $G\in U^*_{n,n-3}$.

(ii) For $k=n-2$, $EuSO(G)\ge 2\left[ \sqrt{13}+\sqrt{6}+\sqrt{19}+(n-5)\sqrt{3}\right]$. Equality holds if and only if $G\in U^*_{n, n-2}$.

In the following theorem, we characterize unicyclic graphs that have the third minimum Euler–Sombor index.

### Theorem 3.7

Let $G\in \mathcal {U}_{n,k}$ ($3\le k \le n-4$) and $G\not \cong U_{n,k}$. Then $EuSO(G)\ge 4\sqrt{19}+2\sqrt{7}+(2n-11)\sqrt{3}$. Equality holds if and only if $G\in U^*_{n,k}$.

### Proof

Let *C* be the unique cycle of length *k* in *G*. Since ($3\le k \le n-4$), at least one of the vertex in *C* is of degree at least 3. Suppose at least 2 vertices in *C* are of degree at least 3, then by Lemmas 3.2 and 3.3, there is a graph $G_1\in U^1_{n,k}$ such that $EuSO(G)\ge EuSO(G_1)$. Thus by Lemma 3.4,

$$EuSO(G)\ge 4\sqrt{19}+2\sqrt{7}+(2n-11)\sqrt{3},$$

and the equality holds if and only if $G\in U^{1}_{n,k}\cap U^{*}_{n,k}$. Now, assume that there is only one vertex, say *v* in *C* of degree at least 3. If $d(v)\ge 4$, then by Lemma 3.2, there exists a graph $G_2\in U^2_{n,k}$ such that $EuSO(G)> EuSO(G_2)$. Thus by Lemma 3.4,

$$EuSO(G)> 4\sqrt{19}+2\sqrt{7}+(2n-11)\sqrt{3}.$$

Otherwise, $d(v)=3$. Since $G\notin U^{1}_{n,k}$, there is another vertex in *G* of degree at least 3. Hence, by Lemma 3.2, there exists a graph $G_{3}\in U^{3}_{n,k}$ such that $EuSO(G)\ge EuSO(G_3)$. So, by Lemma 3.5, $EuSO(G)\ge 4\sqrt{19}+2\sqrt{7}+(2n-11)\sqrt{3}$, and the equality holds if and only if $G\in U^{3}_{n,k}\cap U^{*}_{n,k}$. This completes the proof of the theorem. $\square$

### Remark 3.8

By direct calculation, $EuSO(U_{n,n-1})=2\sqrt{19}+\sqrt{13}+2(n-3)\sqrt{3}$ and $EuSO(C_n)=2n\sqrt{3}$.

The following theorem follows from Theorems 3.1, 3.7 and Remark 3.8.

### Theorem 3.9

(i) If $n=5$, then $EuSO(U^*_{5,3})$ > $EuSO(U_{5,4})$ > $EuSO(U_{5,3})$ > $EuSO(C_5)$. (ii) If $n=6$, then $EuSO(U^*_{6,4})$ > $EuSO(U^*_{6,3})$ > $EuSO(U_{6,5})$ > $EuSO(U_{6,4})$ > $EuSO(C_6)$. (iii) If $n\ge 7$, then $EuSO(U^*_{n,n-2})$ > $EuSO(U^*_{n,n-3})$ > $EuSO(U^*_{n,n-4})=\dots =EuSO(U^*_{n,3})$ > $EuSO(U_{n,n-1})> EuSO(U_{n,3})=\dots =EuSO(U_{n,n-2})$ > $> EuSO(C_n)$.

### Conclusion

This study demonstrates that the Euler–Sombor (EuSO) index is an effective molecular descriptor for QSPR modeling of anticancer drugs. The regression analyses reveal that the EuSO index exhibits strong and statistically significant correlations with key physico-chemical properties, namely boiling point, melting point, enthalpy, and molar refraction. In particular, logarithmic regression models provide superior predictive performance for boiling point, melting point, and molar refraction, while a linear model yields the most reliable predictions for enthalpy.

From a theoretical perspective, this work also advances the study of the Euler–Sombor index in graph theory. By extending the results of Su and Tang^22^, we characterized unicyclic graphs of fixed order and girth that attain the third minimum Euler–Sombor index. This characterization enriches the extremal theory of Sombor-type indices and provides further insight into how structural constraints influence degree-based molecular descriptors.

Overall, the combined QSPR analysis and graph-theoretical investigation highlight the dual significance of the Euler–Sombor index, reinforcing its applicability in chemical graph theory as well as in practical modeling of molecular properties relevant to drug discovery.

## Data Availability

The datasets generated and/or analysed during the current study are available in the ChemSpider repository, [https://share.google/CWCDuNU1J3cBkTXm7].
